# Lanthanum Ferrite Ceramic Powders: Synthesis, Characterization and Electrochemical Detection Application

**DOI:** 10.3390/ma13092061

**Published:** 2020-04-29

**Authors:** Raluca Dumitru, Sorina Negrea, Adelina Ianculescu, Cornelia Păcurariu, Bogdan Vasile, Adrian Surdu, Florica Manea

**Affiliations:** 1 Faculty of Industrial Chemistry and Environmental Engineering, Politehnica University Timisoara, Piata Victoriei No. 2, RO-300006 Timisoara, Romania; sorina.negrea@upt.ro (S.N.); cornelia.pacurariu@upt.ro (C.P.); 2 Department of Oxide Materials Science and Engineering, Faculty of Applied Chemistry and Materials Science, Polytehnic University of Bucharest, Gh. Polizu Street no.1-7, 011061 Bucharest, Romania; a_ianculescu@yahoo.com (A.I.); bogdan.vasile@upb.ro (B.V.); adrian.surdu@upb.ro (A.S.)

**Keywords:** lanthanum ferrite, oxalate precursor, electrochemical detection, capecitabine

## Abstract

The perovskite-type lanthanum ferrite, LaFeO_3_, has been prepared by thermal decomposition of *in situ* obtained lanthanum ferrioxalate compound precursor, LaFe(C_2_O_4_)_3_·3H_2_O. The oxalate precursor was synthesized through the redox reaction between 1,2-ethanediol and nitrate ion and characterized by chemical analysis, infrared spectroscopy, and thermal analysis. LaFeO_3_ obtained after the calcination of the precursor for at least 550–800 °C/1 h have been investigated by X-ray diffraction (XRD), field emission scanning electron microscopy (FE-SEM), transmission electron microscopy (TEM), and high-resolution transmission electron microscopy (HRTEM). A boron-doped diamond electrode (BDD) modified with LaFeO_3_ ceramic powders at 550 °C (LaFeO_3_/BDD) by simple immersion was characterized by cyclic voltammetry and tested for the voltammetric and amperometric detection of capecitabine (CCB), which is a cytostatic drug considered as an emerging pollutant in water. The modified electrode exhibited a complex electrochemical behaviour by several redox systems in direct relation to the electrode potential range. The results obtained by cyclic voltammetry (CV), differential-pulsed voltammetry (DPV), and multiple-pulsed amperometry proved the electrocatalytic effect to capecitabine oxidation and reduction and allowed its electrochemical detection in alkaline aqueous solution.

## 1. Introduction

Perovskite-type oxides have received growing attention due to its diversity and performance [1,2,3,4,5]. Among the perovskite materials, lanthanum ferrite has been investigated intensively due to its potential applications in biosensors [6], (photo)catalysis [7], chemical sensors [8,9], electrochemistry field [10,11,12,13,14,15], magnetic, optical, and ferroelectric properties [16,17], environmentally-friendly pigments [18], spin electronic devices [19,20], and more.

In general, electrochemical behaviors of perovskite-type oxides LaFeO_3_ have been investigated for various applications due to its high oxidation–reduction characteristics and electrical conductivity, e.g., electrocatalysts for oxygen evolution [11], photoelectrochemical water oxidation [12], hydrogen storage [13], gas sensor [14,15,21], voltammetric/amperometric detection of biomolecules [22], solid oxide fuel cells, and electrode materials [23,24].

Many methods have been studied and developed for obtaining LaFeO_3_: sol-gel method [25,26], mechanochemical [27], hydrothermal processes [28], molten salt synthesis [29], combustion method [30,31], microwave-assisted synthesis [32], co-precipitation of hydroxides [33], and polymerizable complex method [34]. Up to date, the precise control of perovskite-type oxides size, shape, and surface is still a challenge. The thermal decomposition of complex compounds precursors represent a simple, efficient, and reliable method for the synthesis of mixed oxides characterized by small particles at a relatively low temperature that depend on the system composition and properties [35,36]. However, it is required to find well-defined redox reaction conditions for precursor generating, which represents the key element to get an effective synthesis process of the mixed oxides characterized through desired advanced properties [37,38]. This is the reason for studying the optimum synthesis conditions (e.g., pH, reaction temperature and time, reactant molar ratio) for each system and corresponding mixed oxides. For example, it is possible to conduct the oxidation of 1,2-ethanediol either glyoxylate anion [39,40] or oxalate anion, in the absence or presence of nitric acid, depending on the working conditions.

Herein, we reported an original method of *in situ* synthesis LaFe(C_2_O_4_)_3_·3H_2_O oxalate compound, as a precursor of LaFeO_3_. This consists of a redox reaction of 1,2-ethanediol with metallic nitrates, in the presence of nitric acid, which was suitable for synthesis of other metallic oxides precursors [37,41,42,43,44,45,46]. The polyheterometallic oxalates, coordinative compounds in which the ligands are tetradentate, are more important because their thermal decomposition generate a large amounts of gases, which leads to nanomaterials with porous structures, high surface area, and homogeneous distribution, which are essential properties for practical sensing applications. The electrocatalytic characteristics in direct relation to the intrinsic morphostructural properties of LaFeO_3_ toward the electrooxidation or electroreduction process of the target analytes are responsible for the performance of the electrochemical detection. Its utility has been reported for gas sensing, e.g., SO_2_, formaldehyde, ethanol [21], and only a few for amperometric/voltammetric of biomolecules, e.g., guanine and uric acid [22], which allows the development of high sensitivity-electrochemical detection.

Capecitabine (CCB), chemically N^4^-pentoxycarbonyl-5’-deoxy-5-fluorocytidine, is a pyrimidine analogue, which acts inside the body as 5-fluorouracil, and commonly used cytostatic drugs in chemotherapy for treating colorectal and breast cancer [47,48]. Since the number of cancer patients has increased considerably, the CCB consumption increased continuously. For example, one-fold from the year 2004 to 2008 in France, and the environmental concentration of 3 ng L^−1^ was reported [49], which led to its presence into the water environment at a higher concentration. Few analytical methods including HPLC (high-performance liquid chromatography), LC-MS (liquid chromatography and mass spectrometry), and LC-MS/MS have been reported for CCB determination in biological samples [50,51,52]. Recently, electrochemical methods have been developed for CCB determination in biological samples using unmodified/modified carbon-based electrode materials, which considered the reduction process of CCB [47,52,53]. It is important to explore new electrode materials and techniques to develop sensitive, fast, simple, and cheap method for CCB determination. In this context, LaFeO_3_ was tested as an electrocatalyst in relation to its electrochemical behavior including the presence of redox systems to allow the CCB detection at LaFeO_3_ modified commercial BDD electrode material. To the best of our knowledge, no study related to CCB detection using LaFeO_3_-based electrode material has been reported. LaFeO_3_-modified boron doped diamond electrode (LaFeO_3_/BDD) was successfully used for the voltammetric and amperometric detection of CCB including both processes of CCB oxidation and reduction.

## 2. Materials and Methods

### 2.1. Materials

Pure LaFeO_3_ was obtained through the calcination in the temperature range of 550 to 800 °C of the LaFe(C_2_O_4_)_3_·3H_2_O precursor, which was obtained previously. It was used as reagents: La(NO_3_)_3_·6H_2_O, (Fe(NO_3_)_3_·9H_2_O, 1,2-ethanediol, 2 M HNO_3_ solution from Merck (Darmstadt, Germany). NaOH and capecitabine (CCB) used was an analytical-grade reagent from Merck. The doubly distilled and deionised water were used for preparing all solutions.

### 2.2. Synthesis of LaFe(C_2_O_4_)_3_·3H_2_O Oxalate Precursor and LaFeO_3_

An aqueous solution containing lanthanum nitrate, iron nitrate, 1,2-ethanediol, and 2 M nitric acid in a molar ratio of 1:1:3:2 that assured the solution pH of 3 was heated for 20 min at about 100 °C in a water bath. The synthesis conditions related to acidic pH of 3 were set-up based on the stoichiometry of the redox reaction related to the molar ratio. The temperature of about 100 °C is aqueous solution boiling temperature and the reaction time of 20 min was considered as the time for consuming reactants. The obtained precursor was purified by washing with acetone and dried in air. VARIAN SpectrAA 110 atomic absorption spectrophotometer (Varian, Palo Alto, CA, USA) was used to determine the metal content and a Carlo Erba 1108 elemental analyzer (Carlo Erba, Milan, Italy) was used for carbon and hydrogen analyzing. LaFeC_6_O_15_H_6_, calc./found: La%: 27.09/27.21, Fe%: 10.89/10.62, C%: 14.04/13.95, and H%: 1.17/1.22. The precursor was calcinated in air under the temperature range from 550 to 800 °C with a heating rate of 10 °C min^−1^ for time duration of one hour to get the LaFeO_3_ ceramic powders.

### 2.3. Characterization of LaFe(C_2_O_4_)_3_·3H_2_O Oxalate Precursor and LaFeO_3_

The comparative Fourier transform infrared spectroscopy (FTIR) spectra of the LaFeO_3_ and oxalate precursor were recorded based on KBr pellets using a Jasco FT-IR spectrophotometer (Jasco, Tokyo, Japan) under the range of 4000–400 cm^−1^. Thermal measurements (TG, DTG, DSC) were carried out in artificial air flow of 20 mL min^−1^ and heating rate of 10 K min^−1^ using a NETZSCH-STA 449C instrument (Netzsch, Selb, Germany) under the temperature range of 25–1000 °C, using alumina crucibles that were performed on the precursor. 

X-ray diffraction (XRD) analyses performed at room temperature by a Rigaku Ultima IV diffractometer (Rigaku Co., Tokyo, Japan), using Ni-filtered CuKα radiation (λ = 1.5418 Ǻ) to characterize the phase purity and crystal structure of calcined powders. To refine the lattice parameters, the Rietveld method using the HighScore Plus 3.0e software (Rigaku Co., Tokyo, Japan) was applied. 

Scanning electron microscopy (FE-SEM), using a high-resolution FEI QUANTA INSPECT F microscope (FEI Co., Eindhoven, The Netherlands) with a field emission gun, was used to assess the size and the agglomeration tendency of the LaFeO_3_ particles. In addition, transmission electron microscopy (TEM/HR-TEM) and selected area electron diffraction (SAED) investigations were performed for a high-accuracy assessment of the morphology and crystallinity degree of the constitutive LaFeO_3_ particles. Moreover, a TecnaiTM G^2^ F30 S-TWIN transmission electron microscope (FEI Co., Eindhoven, The Netherlands) was used to collect the bright-field and high-resolution images.

### 2.4. Electrochemical Detection Application

In order to easily test the electrochemical behaviour of LaFeO_3_ in the presence of capecitabine, the commercial BDD electrode produced by Windsor Scientific Ltd. (Slough, UK). For electroanalytic use, with a boron content of about 0.1, it was modified by simple immersion in 5 mg mL^−1^ LaFeO_3_ suspension. Unmodified and LaFeO_3_-modified boron-doped diamond electrode (LaFeO_3_/BDD) were electrochemically characterized and tested for detection using Autolab Pontentiostat/Galvanostat PGStat 302 (EcoChemie, Utrecht, The Netherlands) controlled with GPES 4.9 software (EcoChemie, Utrecht, The Netherlands) and a classical three-electrode cell. The saturated calomel electrode as a reference (SCE), the platinum electrode as a counter-electrode and the LaFeO_3_ modified/unmodified boron-doped diamond electrode modified (LaFeO_3_/BDD and BDD) as working electrodes were used for the electrochemical applications. The LaFeO_3_/BDD electrode was prepared by simple immersion prior to each electrochemical experiment running. The reproducibility of the modified surface was checked after stabilization by cyclic voltammetry and confirmed by a complete overlay of the cyclic voltammograms.

After electrode immersion, 10 continuous repetitive cyclic voltammograms within the various potential ranges related to each application conditions were applied for the electrochemical stabilization of the electrode. The cyclic voltammetry, differential-pulsed voltammetry, and multiple-pulsed amperometry were applied for the electrochemical characterization and the detection applications.

All measurements were carried out in 0.1 M sodium hydroxide supporting electrolyte at room temperature without a temperature control.

## 3. Results and Discussion

### 3.1. Characterization of LaFe(C_2_O_4_)_3_·3H_2_O Oxalate Precursor

The method of the synthesis of the oxalate complex compound is based on the oxidation reaction of 1,2-ethanediol by the nitrate ion.

3C_2_H_4_(OH)_2_ + (La^3+^ + 3NO_3_^−^) + (Fe^3+^ + 3NO_3_^−^) + 2(H^+^+NO_3_^−^) → LaFe(C_2_O_4_)_3_·3H_2_O_(s)_ + 8NO_(g)_ + 7H_2_O_(g)_ (1)

NO_(g)_ + ½ O_2(g)_ → NO_2(g)_ (2)

**Figure 1:**
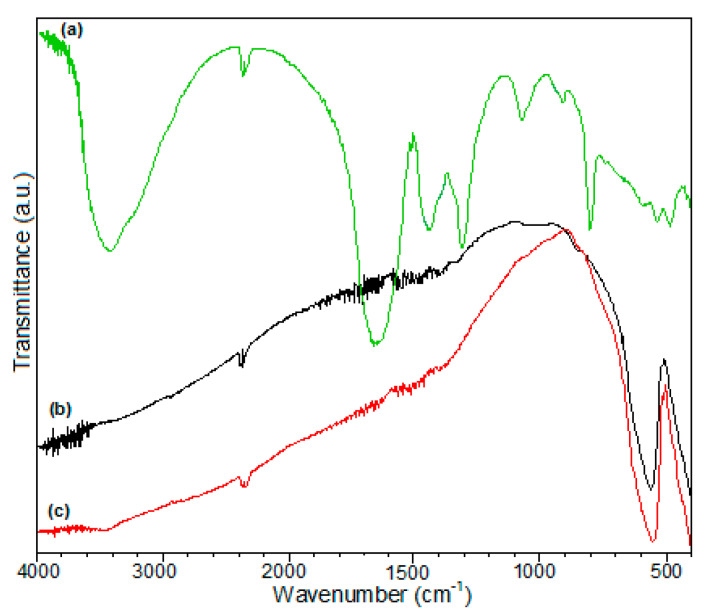
IR vibrational spectra of LaFe(C_2_O_4_)_3_·3H_2_O compound (**a**), LaFeO_3_ 550 °C (**b**)**,** and LaFeO_3_ 700 °C (**c**).

The IR spectrum of oxalate precursor (Figure 1a) reveals the presence of water [3410 cm^−1^ (ν_OH_, ν_H2O_), 794 cm^−1^ (lattice water)] [41,54], oxalate anions [as bidentate ligand: 1642 cm^−1^ (ν_asym (O=C–O)_) + *δ*_sym_ (HOH), 1308 cm^−1^ (ν_sym (O=C–O)_, 1051 cm^−1^ (ν_(C–O)_)] [42]; as tetradentate ligand: 1445 cm^−1^ (ν_sym (OCO)_), 915 cm^−1^ (δ_(OCO)_)] [55], and metal-oxygen linkages [600–400 cm^−1^: ν_(La–O)_ and ν_(Fe–O)_ vibrations]. The IR spectra of LaFeO_3_ obtained after calcination at 550 °C (Figure 1b) and 700 °C (Figure 1c) exhibit only the bands characteristic for vibrations *ν*(La–O) and *ν*(Fe–O) in the range of 600–400 cm^−1^ [43,44]. 

No other bands characteristic to the presence of the water and the oxalate anion can be noticed due to thermal decomposition of the precursor, known as the LaFe(C_2_O_4_)_3_·3H_2_O oxalate compound, which are further detailed and presented.

**Figure 2:**
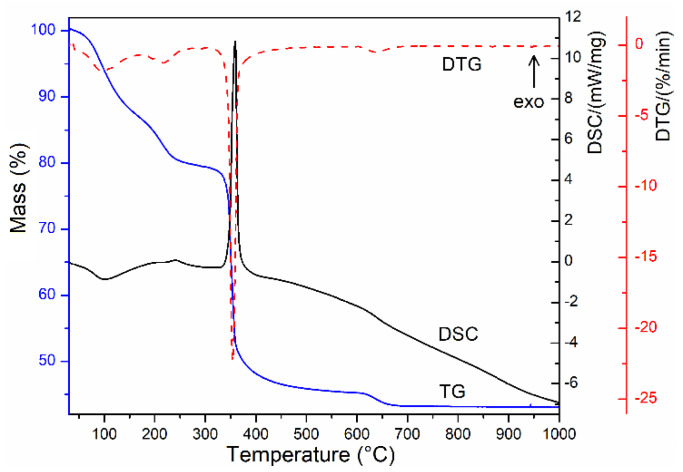
Thermal curves TG (thermogravimetric), DTG (derivative thermogravimetric), and DSC (differential scanning calorimetry) of LaFe(C_2_O_4_)_3_·3H_2_O compound.

La(III)-Fe(III) oxalate trihydrate decomposes (Figure 2) via three steps with the formation of a carbonate intermediate.

The first step of thermal decomposition of LaFe(C_2_O_4_)_3_·3H_2_O (25–160 °C, mass loss: found 11.10%, calcd. 10.53%) is associated with an endothermic effect, and the mass loss is attributed to the evolving of the lattice water [43].

The next decomposition step (160–280 °C) characterized by an endothermic effect is due to the degradation of the terminal oxalate anions (mass loss, found 10.40%, calcd. 10.92%) with the formation of a carbonate intermediate, [CO_3_La(C_2_O_4_)FeCO_3_] [44,45].

The strong exothermic effect that characterizes the third step (280–550 °C) is attributed to the oxidative degradation of ${\mathrm{CO}}_{3}^{2-}$ and the last $C_{2}O_{4}^{2-}$ with the formation of the lanthanum ferrite, which retains small amounts of carbon dioxide (mass loss: found 34.03%, calcd. 34.32%). In the temperature range of 550–700 °C, the mass loss corresponds to the removal of carbon dioxide. 

Simultaneously, with this endothermic decomposition step, an exothermic process of crystallization, involving the formation of the perovskite skeleton from an amorphous phase also takes place, so that, only a small and flattened endothermic peak, as a resultant of the two opposite processes, can be observed on the DSC curve [45,46]. 

The thermal analysis suggests the following sequence for decomposition of LaFe(C_2_O_4_)_3_·3H_2_O in static air atmosphere.

(3) $$\mathrm{LaFe}(C_{2}O_{4}{)}_{3}\cdot 3H_{2}O_{\left(s\right)}\;\overset{\left(I\right)}{\to }3\;H_{2}O_{\left(g\right)}\;+\;\mathrm{LaFe}(C_{2}O_{4}{)}_{3(s)}$$

(4) $$\mathrm{LaFe}(C_{2}O_{4}{)}_{3(s)}\;\overset{\left(\mathrm{II}\right)}{\to }\;2\;CO_{\left(g\right)}+{\mathrm{CO}}_{3}{\mathrm{LaC}}_{2}O_{4}\mathrm{FeC}O_{3(s)}$$

(5) $${\mathrm{CO}}_{3}{\mathrm{LaC}}_{2}O_{4}\mathrm{FeC}O_{3(s)}\;\overset{+1/2O_{2}\;(\mathrm{III})}{\to }\;4\;{\mathrm{CO}}_{2(g)}+{\mathrm{LaFeO}}_{3(s)}$$

### 3.2. Characterization of LaFeO_3_ Powders

**Figure 3:**
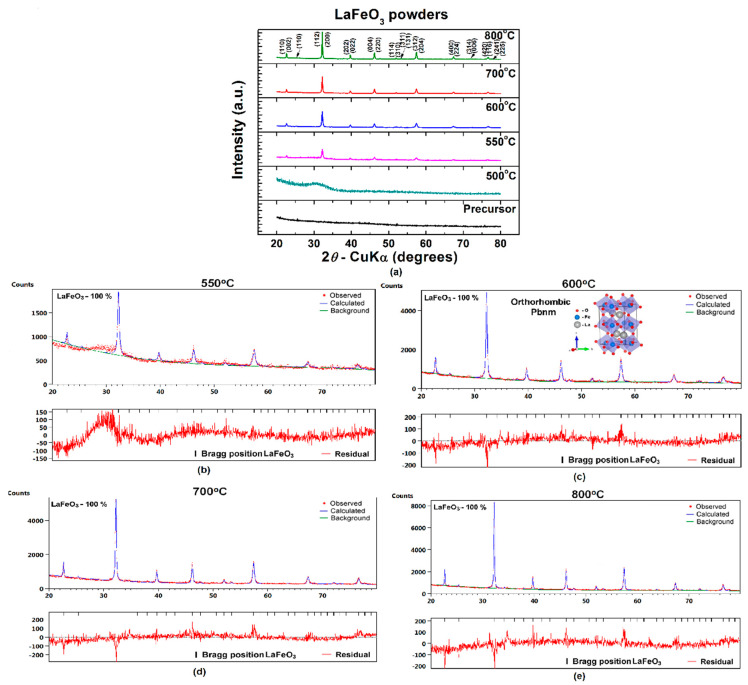
(**a**) Room-temperature XRD patterns and (**b**–**e**) results of Rietveld analysis of XRD data for LaFeO_3_ powders calcined at various temperatures: (**b**) 550, (**c**) 600, (**d**) 700, and (**e**) 800 °C.

The room-temperature X-ray diffraction patterns presented in Figure 3a show that the as-prepared precursor, as well as the powder that resulted after calcination at 500 °C, are amorphous, while, in the powders obtained at higher temperatures (*T* ≥ 550 °C), the main reflections specific to the LaFeO_3_ perovskite phase with orthorhombic *Pbnm*(62) structure were detected. For the powder calcined at 550 °C, the broader profile and the lower intensity of the diffraction peaks as well as the higher background of the corresponding diffraction pattern suggested that a significant amount of an amorphous phase still persists in the sample. The increase of the annealing temperature from 550 to 800 °C induces the reduction of the amount of amorphous phase, concurrently with the gradual increase of the crystallintiy degree and purity of the perovskite phase reflected in the enhancement of intensity of the main diffraction lines of LaFeO_3_.

The results of the Rietveld analysis of the room temperature diffraction data of LaFeO_3_ powders thermally treated at various temperatures in the temperature range of 550–800 °C are presented in Figure 3b–e. The quality of the samples is indicated by the parameters provided by the Rietveld refinement, *R* expected (*R*_exp_), *R* profile (*R*_p_), weighted *R* profile (*R*_wp_), and goodness of fit (*χ*^2^), which show values in good agreement with other literature data [56] (Table 1). 

**Figure 4:**
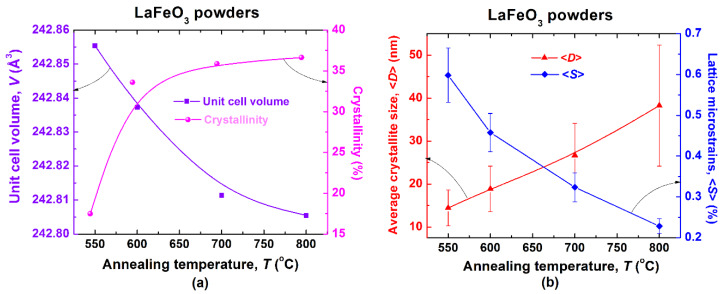
Annealing temperature dependence of: (**a**) unit cell volume and crystallinity and (**b**) average crystallite size and lattice microstrains.

The dependence of the lattice parameters, *a*, *b*, and *c* and, consequently, of the unit cell volume *V*, on the calcination temperature, exhibits a decreasing trend (Table 1). The progress of the crystallization process involves a clear contraction of the unit cell, as shown in Figure 4a. 

Even if the coarsening process evolves with the increase of annealing temperature, the values of the average crystallite size, also determined from the XRD data, are kept in the nanometric range, varying from 17.5 nm for the LaFeO_3_ powder calcined at 550 °C to 36.7 nm in the case of the powder calcined at 800 °C. The significantly lower rate of increasing both the proportion of crystalline phase with respect to the amount of amorphous phase and the crystallite size with the temperature increase for LaFeO_3_ particles investigated in this study, in comparison to the steeper increase of these characteristics in the case of BiFeO_3_ powders calcined in the same temperature range reported earlier, which can be related to the higher refractoriness of lanthanum relative to that of Bi in the ferrite-type compounds [44]. As expected, the increase of the crystallite size determines a lattice relaxation proved by the clear decrease in the lattice micro-strains when increasing the calcination temperature (Figure 4b). Therefore, the increase of the crystallite size involves the reduction in the inter-atomic spacing, which results in the contraction of the unit cell.

**Figure 5:**
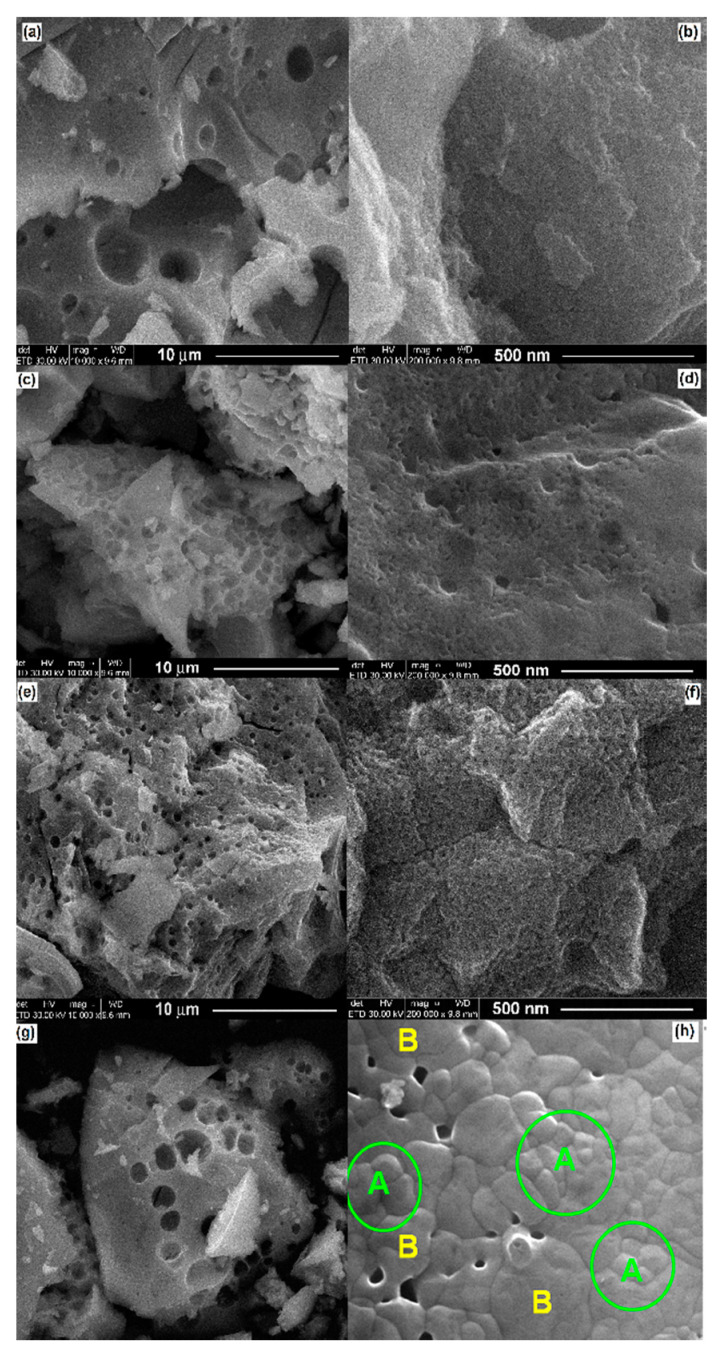
FE-SEM overall (**a**,**c**,**e**,**g**) and detail (**b**,**d**,**f**,**h**) images of LaFeO_3_ powders calcined at various temperatures: (**a**,**b**)—550 °C, (**c**,**d**)—600 °C, (**e**,**f**)—700 °C, and (**g**,**h**)—800 °C (green circles A—groups of nano-sized grains, yellow B—larger grains formed by the coalescence of the smaller ones).

For all the powders under investigation, the FE-SEM analyses showed the clear tendency of the LaFeO_3_ particles to form large (of few tens of microns), non-uniform (as shape and size) aggregates, with a spongeous aspect, as indicated by the FE-SEM overall images of Figure 5a,c,e,g. 

The higher magnification FE-SEM images have taken in order to notice the structuring inside the aggregates showed that the powder calcined at 550 °C consists of very small-sized particles (most of them below 15 nm). The value of the average particle size is difficult to be estimated mainly because of the presence of a significant amount of amorphous phase (as XRD data indicated), which impeded the clear distinction of the particle’s boundaries (Figure 5b). The structuring inside the aggregates becomes better highlighted in the case of the powders that resulted after annealing at 600 and especially at 700 °C (Figure 5d,f). The presence of the amorphous phase is still noticed along with the crystalline aggregates in the LaFeO_3_ powder calcined at 600 °C (Figure 5d), while the aggregates of the powder calcined at 600 °C seems to consist of porous agglomerates of 300–500 nm, inside which nanometric particles (of 25–35 nm), linked together by necks and form 3D networks are observed (Figure 5f). Taking into account the values of the average crystallite size determined from the XRD data, one can assume that the nanoparticles of the LaFeO_3_ powders resulted after annealing in the temperature range of 550–700 °C are single crystals. The FE-SEM detail image of Figure 5h indicates that the LaFeO_3_ powder calcined at 800 °C consists of partially-sintered blocks, formed of nano-sized crystalline grains (with an average grain size <*DG*> = 69.4 nm) with well-defined boundaries and triple junctions, between which a small amount of intergranular porosity is detected. In some regions, the grain growth process, favored by the higher annealing temperature, induced a coalescence of some smaller grains, with the formation of the larger ones (of 200–300 nm) as well as the concurrent modification of some intergranular pores into intragranular pores (Figure 5h). Considering the value of the average crystallite size obtained from the XRD data, the values of the grain/particle size inside the agglomerates estimated from the FE-SEM observations suggest that the grains of the LaFeO_3_ powder calcined at 800 °C exhibit a polycrystalline nature. Thus, we were able to estimate that the smaller grains consist of 2–3 crystallites, while the larger ones are formed from 6–10 crystallites.

**Figure 6:**
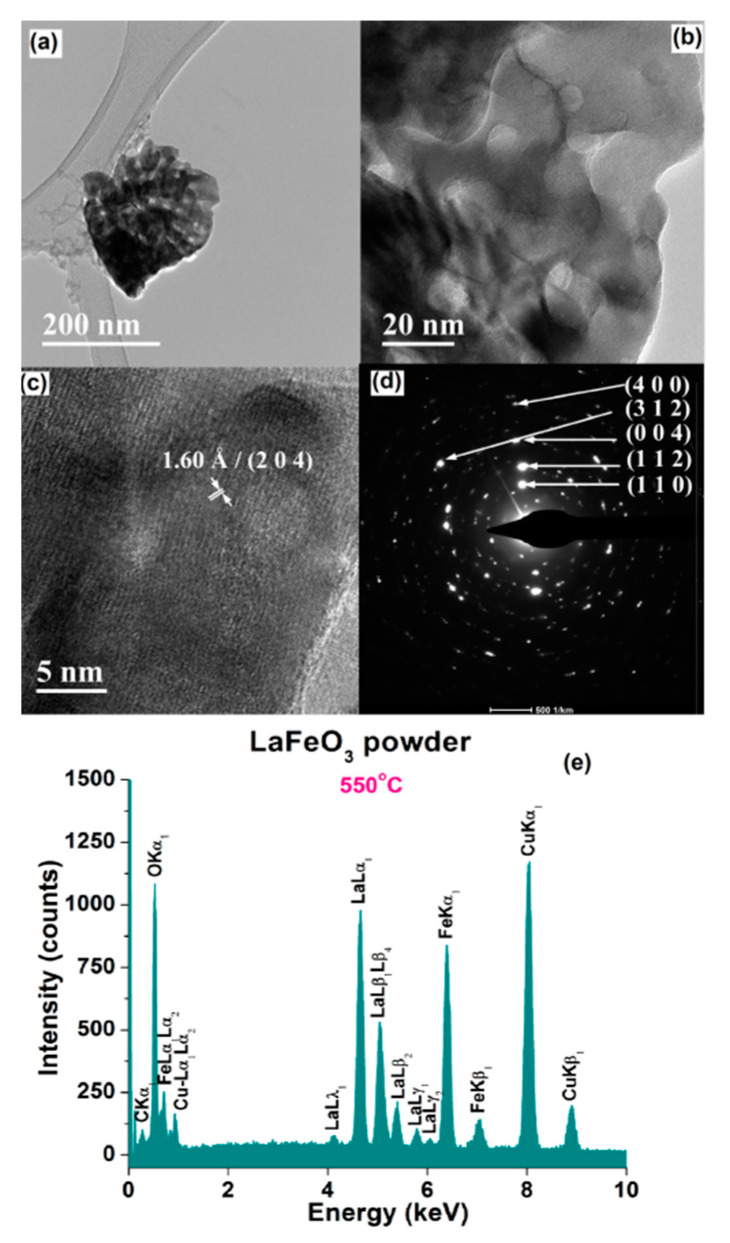
(**a**,**b**) TEM images of various magnifications: (**c**) HRTEM image, (**d**) SAED pattern, and (**e**) EDX spectrum for LaFeO_3_ powder calcined at 550 °C.

**Figure 7:**
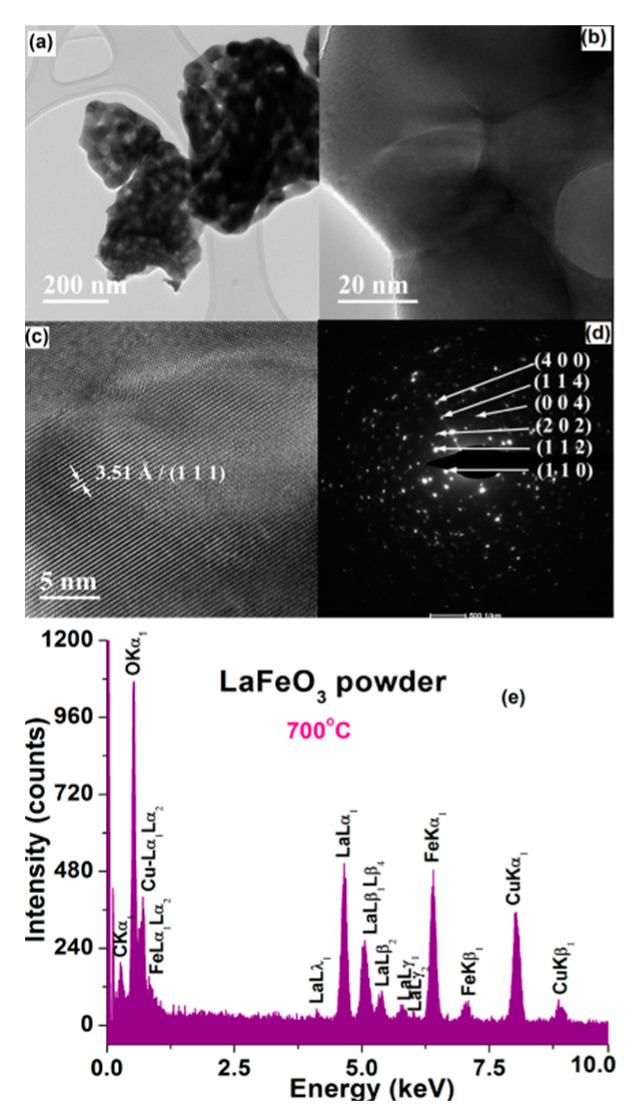
(**a**,**b**) TEM images of various magnification, (**c**) HRTEM image, (**d**) SAED pattern, and (**e**) EDX spectrum for LaFeO_3_ powder calcined at 700 °C.

The FE-SEM observations are sustained by the results of TEM investigations. Thereby, the lower magnification TEM images of Figure 6a and Figure 7a clearly indicate the presence of the submicronic aggregates of various sizes and with irregular shapes. The higher magnification TEM images emphasized the structuring of the aggregates, which are formed by particles of 10–20 nm in the case of the powder calcined at 550 °C (Figure 6b) and of ~40–50 nm for powder calcined at 700 °C (Figure 7b).

The HRTEM image of the LaFeO_3_ sample calcined at 550 °C indicate the presence of some small particles with sizes of 6.5–10 nm. Inside these particles, oriented fringes spaced at 1.60 Å corresponding to the crystalline plane (2 0 4) were noticed (Figure 6c). It is difficult to notice these small particles because they are usually embedded into an amorphous matrix. In the case of the powder calcined at 700 °C, the lower amount of amorphous phase allowed to better visualize the long-range ordered fringes, so that the HRTEM image clearly emphasizes long-range ordered fringes spaced at 3.51 Å, corresponding to the crystalline plane (1 1 1) (Figure 7c).

For both powders, the high crystallinity degree of the randomly oriented particles is also pointed out by the bright spots, forming concentric diffraction rings, assigned to several crystalline planes of the perovskite LaFeO_3_ phase in the specific SAED (selected area electron diffraction) patterns of Figure 6d and Figure 7d.

EDX (energy-dispersive X-ray analysis) analyses were also performed in order to determine the elemental composition of the LaFeO_3_ powders under investigation. The EDX spectra show only the presence of La, Fe, and O species, which indicates that no contamination took place during the powders processing (Figure 6e and Figure 7e). 

The morphostructural properties of the LaFeO_3_ are different functions of the calcination temperature, and the lowest particles were obatined at a relatively low temperature of 550 °C in comparison with other materials synthesized by the same method [43,44,45,46].

### 3.3. Electrochemical Characterization and Detection Application

**Figure 8:**
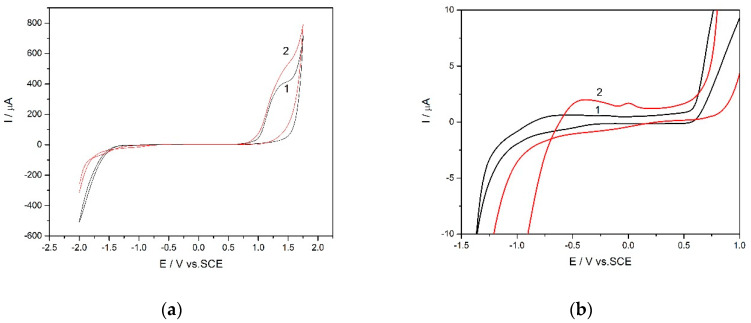
(**a**) Cyclic voltammetry recorded at an unmodified BDD electrode (curve 1) and LaFeO_3_/BDD electrode (curve 2) in 0.1 M NaOH supporting the electrolyte, scan rate of 0.05 Vs^−1^. (**b**) Detail of cyclic voltammetry recorded at an unmodified BDD electrode (curve 1) and LaFeO_3_/BDD electrode (curve 2) in 0.1 M NaOH supporting electrolyte.

Based on the morphostructural properties of LaFeO_3_ ceramic powders, which were presented above and discussed in relation with the electroactivity capacity and electro-catalytical effect, for testing in electrochemical detection application LaFeO_3_ synthesized at 550 °C. The comparative electrochemical behaviours of unmodified and LaFeO_3_-modified BDD electrodes in alkaline medium was studied by cyclic voltammetry (CV) and the results are presented in Figure 8a,b. It can be noticed that the oxidation and coupled reduction peak for LaFeO_3_/BDD, which can be attributed to Fe^2+/^Fe^3+^ and Fe^3+^/Fe^4+^ redox systems according to the literature [12]. In addition, capacitive component of the current is much larger in comparison with the unmodified BDD electrode, which is expected for the electrocatalyst electrode material. Thus, a depolarization effect toward the oxygen evolution reaction and a polarization effect on the hydrogen evolution reaction were manifested through LaFeO_3_ on the BDD electrode surface. The electrocatalytic activity of the LaFeO_3_ sample is relevant with the crystalline size and the rate of oxygen migration from bulk towards the surface [57,58], which relates to the applied potential range. 

**Figure 9:**
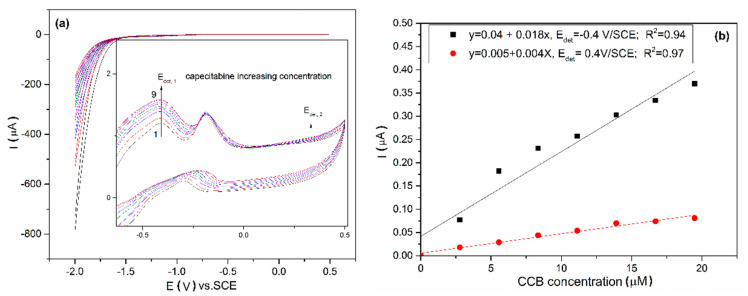
(**a**) Cyclic voltammetry recorded at LaFeO_3_/BDD electrode in 0.1 M NaOH (curve 1) and in the various CCB concentrations ranged from 2.5 µM to 22.5 µM (curves 2–9), scan rate of 0.05 Vs^−1^. (**b**) Calibration plots of anodic peak with various CCB concentrations ranged from 2.5 µM to 22.5 µM (curves 2–9).

Both unmodified and LaFeO_3_/BDD electrodes were tested in the presence of 5 µM CCB and no signal was found at an unmodified BDD electrode (the results are not shown here). The behavior of the LaFeO_3_/BDD electrode in the presence of various CCB concentrations ranged from 2.5 to 22.5 µM is shown in Figure 9a and it can be noticed that the current grows with CCB concentration increasing for two potential values, which can be considered as detection potentials. The redox systems manifested to −0.4 V/SCE and +0.4 V/SCE showed a strong promoting effect and high stability towards the electrochemical oxidation of CCB. The linear dependence between the current and CCB concentration was found at the potential value of −0.4 V/SCE and 0.4 V/SCE (Figure 9b). Under this potential range, no increase of any cathodic reduction peak was noticed for the LaFeO_3_/BDD electrode, which informed that the CCB oxidation is not reversible.

**Figure 10:**
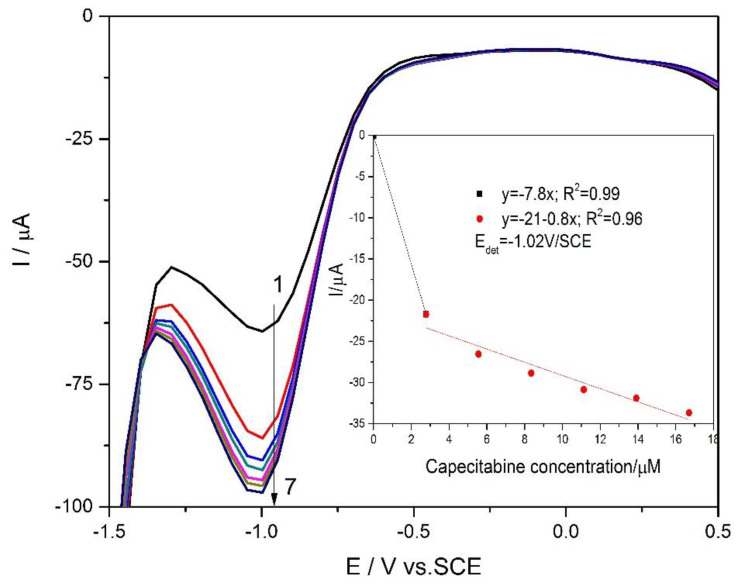
Differential-pulsed voltammograms recorded at LaFeO_3_/BDD electrode in 0.1 M NaOH (curve 1) and in the various CCB concentrations ranged from 2.5 µM to 17 µM (curves 2–7), scan rate of 0.05 Vs^−1^, modulation amplitude of 0.2 V, and step potential of 0.05 V.

Taking into account that differential-pulsed voltammetry technique (DPV) exhibit the advantages of the higher sensitivity in comparison with CV, this technique was tested in various working conditions related to modulation amplitude and the step potential. The detection results depended by the direction of the applied potential scanning. No reproducible results were achieved for anodic scanning, which suggest that this technique is not appropriate for the CCB detection based on its oxidation process. However, very good results were reached by potential scanning in a cathodic sense under a modulation amplitude of 0.2 V and step potential of 0.05 V (Figure 10). Cathodic peak characteristics to CCB reduction was found at the potential value of −1.02 V/SCE, which should be due to the oxidation-based activation of the electrode surface. The cathodic peak current increased linearly with CCB concentration for two concentration ranges below 2.5 µM CCB and ranged from 2.5 µM to 17 µM CCB (see inset of Figure 10).

**Figure 11:**
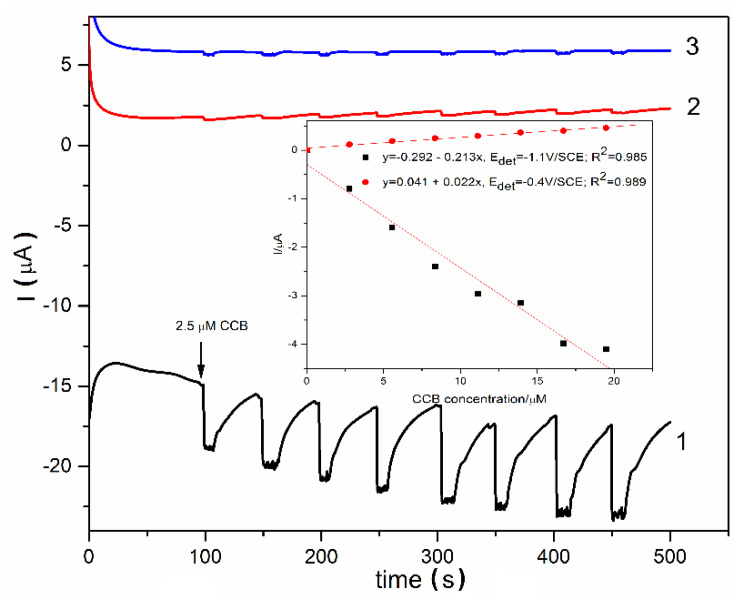
Multiple-pulsed amperograms (MPAs) recorded at the LaFeO_3_/BDD electrode in a 0.1 M NaOH supporting electrolyte and adding 2.5 µM CCB concentrations at three potential levels applied for time duration of 50 ms: −1.1 V/SCE (curve 1), −0.4 V/SCE (curve 2), and 0.4 V/SCE (curve 3). Inset: Calibration plots of current vs CCB concentrations for −1.1 V/SCE and −0.4 V/SCE.

One of the most desired technique for electro-detection is chronoamperometry (CA) due to its simplicity and easy application, which was tested for the LaFeO_3_/BDD electrode in CCB detection. A very low signal was achieved (the results are not shown here), which led to testing three-levels potential based multiple-pulsed amperometry (MPA) as a variant of CA. The potential levels were selected based on the results of voltammetry techniques of both CV and DPV, considering the oxidation and reduction processes related to CCB. According to the literature [59], the oxidation steps of the CCB to 5″-deoxy-5-fluorocytudine and further to five fluorouracil and its reduction to dihydrofluorouracil should be considered based on the previous results of CV and DPV related to the potential range and scanning sense. Amperograms recorded by MPA are presented in Figure 11. The pulses were applied continuously using the following scheme:

(a) −1.1 V/SCE for a duration of 50 ms, where CCB reduction occurred,

(b) −0.4 V/SCE for a duration of 50 ms, where CCB oxidation occurred involving Fe^2+^/Fe^3+^ redox system,

(c) 0.4 V/SCE for a duration of 50 ms, where further CCB oxidation occurred involving Fe^3+^/Fe^4+^ redox system besides an oxygen evolution reaction starting.

The electroanalytical parameters for CCB detection related to sensitivity, the lowest limit of detection, and the limit of quantification determined for each above-mentioned technique are gathered in Table 2. 

In comparison with reported results related to CCB electrochemical detection presented in Table 3, it can be noticed that the superiority of this electrode in relation with a very good limit of detection (LOD) and considering its availability for either CCB oxidation or CCB reduction with the mention that the literature reported only the detection procedure based on CCB reduction.

## 4. Conclusions

In the present work, LaFeO_3_ powders were synthesized using a new method, based on the thermal decomposition of in situ obtained LaFe(C_2_O_4_)_3_·3H_2_O compound.

Single phase LaFeO_3_ powders with orthorombic *Pbnm* perovskite structure were obtained after annealing at temperatures ranging between 550 and 800 °C. The crystallinity degree reflected in the values of the average crystallite size increases as the annealing temperature increased. Concurrently, a contraction of the unit cell volume is induced by the increase of the annealing temperature. Small particles, with sizes below 20 nm and with a high aggregation tendency, were obtained after annealing at 550 °C. Even if the increase of the annealing temperature at 800 °C induces a coarsening process, the size of the LaFeO_3_ particles was kept in the nanometric range (~70 nm). However, a clear tendency to form partially-sintered blocks due to the coalescence of the small particles into larger, polyhedral grains with well-defined boundaries was noticed for the LaFeO_3_ sample calcined at 800 °C.

The LaFeO_3_/BBD electrode exhibited the electrocatalytic activity toward the capecitabine (CCB) oxidation and reduction depending on the applied potential range and scanning direction that influenced the iron redox systems, considered as a basis for developing detection methods. Cyclic voltammetry (CV) and differential-pulsed voltammetry (DPV) techniques operated under specific working conditions allowed us to develop the CCB voltammetric detection method. The best analytical performance was reached using DPV based on the CCB reduction mechanism. Multiple-pulsed amperometry operated under three potential levels implied CCB oxidation and a reduction mechanism, which allows CCB detection in the same time at the two potential values of −1.1 V/SCE and −0.4 V/SCE corresponding to CCB reduction and respective oxidation. The results of electroanalytical performance for CCB detection and its flexibility for anodic and/or cathodic detection made the LaFeO_3_/BDD electrode have great potential for selective or simultaneous detection of CCB in aqueous matrix.

## Figures and Tables

**Table 1:** Rietveld refined structural parameters for LaFeO_3_ powders annealed at various temperatures.

Compound		LaFeO_3_			
Annealing Temperature (°C)		550	600	700	800
Crystal System		Orthorhombic (ICCD no. 04-013-6775)			
Space Group		Pbnm (62)			
Lattice parameters (Å)	*A*	5.562 ± 0.011	5.562 ± 0.003	5.561 ± 0.002	5.561 ± 0.002
	*B*	7.856 ± 0.016	7.856± 0.004	7.856 ± 0.003	7.856 ± 0.002
	*C*	5.558 ± 0.012	5.558 ± 0.003	5.558 ± 0.002	5.558 ± 0.001
Unit cell volume, *V* (Å^3^)		242.9	242.8	242.8	242.8
*R* factors (%)	*R*_exp_	5.520	5.612	5.892	5.664
	*R*_p_	5.244	4.814	5.488	5.041
	*R*_wp_	6.416	5.845	6.578	6.057
	*χ* ^2^	1.351	1.085	1.246	1.143

**Table 2:** The electroanalytical parameters for CCB determination using LaFeO_3_/BDD electrode.

Technique	Mechanism Detection	Detection Potential/ V vs. SCE	Sensitivity/ µA µM^−1^ cm^−2^	Correlation Coefficient/R^2^	LOD */µM	LQ/µM	RSD ** %
CV	CCB oxidation	−0.40	0.257	0.971	0.021	0.038	0.10
		0.40	0.050	0.966	0.590	1.970	1.02
DPV	CCB reduction	−1.02	111.0 (CCB concentration lower than 2.5 µM);	0.990	0.010	0.250	2.50
			11.43 (CCB concentration higher than 2.5 µM)	0.960	0.734	2.446	2.50
MPA	CCB reduction and oxidation	−1.1	3.040	0.985	0.158	0.527	0.84
		−0.4	0.314	0.989	0.103	0.343	0.68

* The limit of detection was measured under 3 Sb/m. ** relative standard deviation was determined for three replicates.

**Table 3:** Comparison of the limit of detection for CCB electrochemical detection using a different electrode material.

Electrode	Limit of Detection/µM	Reference
Glassy carbon	0.113	[47]
ZnO/MWCNT/CPE	0.030	[50]
AuNPs/SGNF/GCE	0.017	[52]
LaFeO_3_/BDD	0.010	This work
